# *Candida africana* and *Candida dubliniensis* as causes of pediatric candiduria: A study using *HWP1* gene size polymorphism

**DOI:** 10.3934/microbiol.2020017

**Published:** 2020-08-25

**Authors:** Bahram Nikmanesh, Kazem Ahmadikia, Muhammad Ibrahim Getso, Sanaz Aghaei Gharehbolagh, Shima Aboutalebian, Hossein Mirhendi, Shahram Mahmoudi

**Affiliations:** 1 Department of Medical Laboratory Sciences, School of Allied Medical Sciences, Tehran University of Medical Sciences, Tehran, Iran; 2 Department of Medical Parasitology and Mycology, School of Public Health, Tehran University of Medical Sciences, Tehran, Iran; 3 Department of Medical Microbiology and Parasitology, Faculty of Clinical Sciences, College of Health Sciences, Bayero University Kano, PMB 3011, Kano-Nigeria; 4 Department of Medical Parasitology and Mycology, School of Medicine, Isfahan University of Medical Sciences, Isfahan, Iran; 5 Department of Medical Parasitology and Mycology, School of Medicine, Iran University of Medical Sciences, Tehran, Iran

**Keywords:** Candiduria, *Candida albicans* complex, *Candida africana*, *Candida dubliniensis*, pediatrics

## Abstract

**Background:**

*Candida* species cause a wide spectrum of disease entities. *Candida africana* and *Candida dubliniensis*–members of *Candida albicans* complex-are currently gaining both clinical and epidemiologic significance.

**Materials and methods:**

Totally, 150 pediatric isolates that had previously been identified as *C. albicans* species complex based on a positive germ tube test were included. The isolates were cultured on CHROMagar *Candida* medium to ensure their purity and the results of germ tube test. For definitive speciation, PCR amplification and size polymorphism of the *hyphal wall protein 1* (*HWP1*) gene was used. The results of *HWP1*-PCR were confirmed by sequencing the amplified fragments for randomly selected isolates of *C. africana* and *C. dubliniensis*.

**Results:**

All 150 isolates included in this study were reconfirmed as *C. albicans* complex on chromogenic media. Based on the *HWP1* gene size polymorphism, 141 (94%) isolates were identified as *C. albicans*, 2 (1.33%) as *C. africana*, and 1 (0.67%) as *C. dubliniensis*. The remaining 6 (4%) *C. albicans* complex isolates were a mix of *C. albicans* and *C. africana*. All isolates of *C. africana* and *C. dubliniensis* have been recovered from cases of candiduria.

**Conclusion:**

*C. africana*, either alone or mixed with *C. albicans*, could be a cause of candiduria among pediatric patients and should not be ignored.

## 1. Introduction

The frequency of fungal infections is increasing worldwide. Species of the genus *Candida* are opportunist fungi capable of causing mild cutaneous to severe and life-threatening systemic infections in susceptible individuals. Among these species, *Candida albicans* is the most clinically encountered pathogen and most researched [1],[2]. Other species, such as *Candida africana* and *Candida dubliniensis*-closely related to *C. albicans*-initially referred to as ‘atypical’ *Candida* species are now fully characterizable using molecular techniques and classified as distinct species in *C. albicans* species complex [3]–[6]. Of recent, the frequency of ‘atypical’ *Candida* species among clinical *Candida* isolates has been rising [7],[8]. *C. africana* infections have been reported from various countries and genitourinary tract samples, especially vaginal swabs have been found as the most common source of isolation [5],[9]–[12]. Earlier, *C. dubliniensis* was mainly isolated from oral cavity of HIV/AIDS patients but subsequent epidemiological studies revealed that this species is recoverable from other body sites such as skin, vagina, respiratory tract, and urine of both HIV-infected and non-HIV patients [13]–[16]. Both *C. africana* and *C. dubliniensis* are clinically important members of *C. albicans* complex but their identification using conventional techniques proves to be challenging due to mutual sharing of similar morphological characteristics. Molecular tools suitable for identification of *Candida* species include the sequence-analysis of the ribosomal DNA (rDNA) ITS region; the use of duplex/multiplex PCR for identification of isolates such as *C. albicans* and *C. dubliniensis*; and amplification of the *hyphal wall protein 1* (*HWP1*) gene - the first molecular method used to discriminate *C. dubliniensis* and *C. africana* from *C. albicans* [4]. Molecular studies based on the ITS data sequence revealed that *C. africana* is a separate species from *C. albicans* with an interspecies sequence homology of 99.3 to 99.8%; whereas *C .dubliniensis* differs from *C. albicans* with an interspecies sequence homology of 91.2–94.4% [17]. Despite their clinical significance, they are rarely sought for in routine laboratory examination-many members of the complex could be misidentified in less suspicious cases-due to their low frequencies in clinical settings [18]. In this study, we aimed to identify the minor species (*C. africana* and *C. dubliniensis*) in the *C. albicans* complex recovered from different clinical specimens using *HWP1* gene amplification method among pediatric patients at a tertiary medical center in Tehran, Iran.

## 2. Material and methods

### 2.1. *Candida albicans* complex isolates

One hundred and fifty clinical isolates of *C. albicans* species complex were included in this study. These isolates had been collected during routine work at the microbiology laboratory of Children's Medical Center, Tehran, Iran. The isolates had previously been identified as *C. albicans* species complex based on a positive result in germ tube test.

### 2.2. Re-identification of isolates

To confirm the purity of isolates and the results of germ tube test, the isolates were streaked on CHROMagar *Candida* medium (CHROMagar, France). The results of germ tube test were confirmed if characteristic green colonies grown on the medium.

### 2.3. Molecular identification

The isolates were investigated based on the size polymorphism of the *HWP1* gene using primers CR-f (GCT ACC ACT TCA GAA TCA TCA TC) and CR-r (GCA CCT TCA GTC GTA GAG ACG). PCR was performed using an initial denaturation at 95 °C or 5 minutes; 35 cycles of denaturation at 94 °C for 45 seconds, annealing at 58 °C for 40 seconds, and extension at 72 °C for 55 seconds; and a final extension at 72 °C for 10 minutes [4]. To fasten the procedure of molecular identification, we used a colony-PCR approach instead of extracting the genomic DNA and using it as template. Briefly, using sterile micropipette tips, an overnight colony of each isolate was transferred into a PCR reaction tube containing 12.5 µL of 2X master mix (Amplicon, Denmark), 1 µL of forward and reverse primers (10 pg/µL), and 10.5 µL of deionized, sterile distilled water. PCR amplicons were visualized using a transilluminator (Syngene, USA) after electrophoresis in 1.5% w/v agarose gel. The PCR product sizes of 941 bp, ∼700 bp, and 569 bp were identical to *C. albicans*, *C. africana*, and *C. dubliniensis*, respectively.

To confirm the results of *HWP1* size polymorphism, isolates of *C. africana* and *C. dubliniensis* were randomly selected and a fragment of the *HWP1* gene was amplified using the same primers and PCR conditions described earlier. The PCR products were subjected to sequencing (Macrogene, South Korea) and the results were analyzed using Basic Local Alignment Search Tool (BLAST) in the NCBI database (http://blast.ncbi.nlm.nih.gov/Blast).

## 3. Results

**Figure 1.**
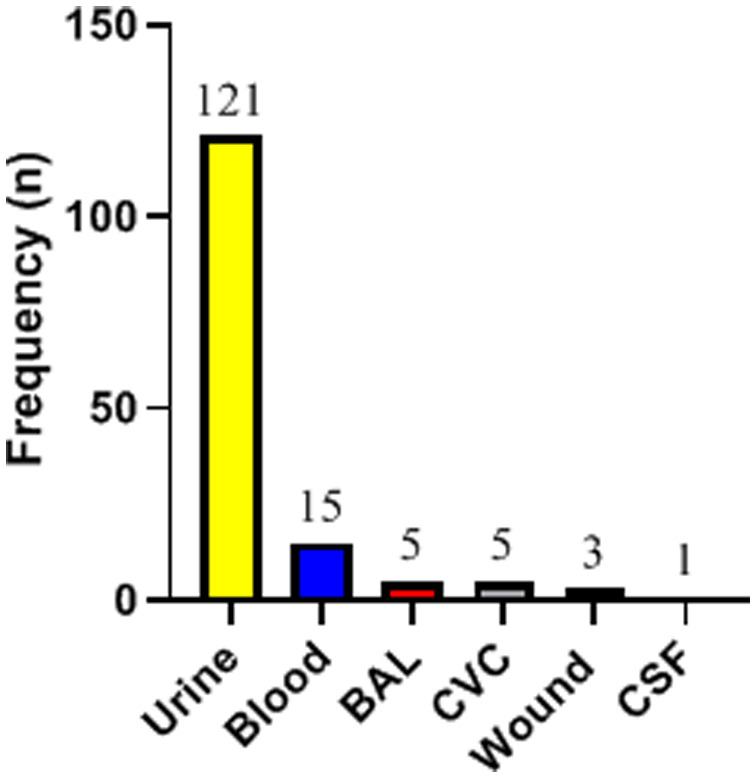
Distribution of 150 *Candida albicans* complex isolates based on the specimen type (BAL: bronchoalveolar lavage; CVC: central venous catheter; CSF: cerebrospinal fluid).

The isolation source of the 150 *C. albicans* species complex isolates is shown in Figure 1. All 150 isolates included in this study were reconfirmed as *C. albicans* complex on chromogenic media by growing as green colonies. By molecular characterization using *HWP1* gene size polymorphism, 141 (94%) isolates were identified as *C. albicans*, 2 (1.33%) isolates as *C. africana*, and 1 (0.67%) isolate as *C. dubliniensis*. The remaining 6 (4%) *C. albicans* complex isolates were found to be a mix of *C. albicans* and *C. africana*. Accordingly, in total, 156 isolates were identified from 150 *C. albicans* complex isolates, of which 147 (94.23%) were *C. albicans*, 8 (5.13%) were *C. africana*, and 1 (0.64%) was *C. dubliniensis*. Two pure *C. africana* isolates and the *C. dubliniensis* isolate were subjected to PCR-sequencing and the obtained results confirmed their identity. The sequences were deposited in GenBank under accession numbers MT829308–MT829310. Regarding the source of isolation, all isolates (pure and mixed) of *C. africana* and the isolate of *C. dubliniensis* were recovered from urine specimens. Table 1 shows the distribution of various species based on the isolation source.

**Table 1.** Distribution of *Candida* albicans complex isolates based on the isolation source.

Specimen	*Candida* species (n)			
	*C. albicans*	*C. africana*	*C. dubliniensis*	Mix of *C. albicans* and *C. africana*
Urine	112	2	1	6
Blood	15			
Central venous catheter	5			
Bronchoalveolar lavage	5			
Wound discharge	3			
Cerebrospinal fluid	1			
Total isolates	141	2	1	12*

* Including 6 *C. africana* and 6 *C. albicans* isolates.

## 4. Discussion

The ‘*C. albicans* complex’ is a group of opportunistic fungal pathogens responsible for more than 50% of candidiasis [1],[7]. Species of this group are more often involved in oral and vaginal human infections [9],[10],[12],[19]. However, infections from other body sites such as respiratory tracts, urinary tracts, wounds, and skin, have been reported [12],[13],[20],[21]. *Candida* infection is a common phenomenon in pediatric subpopulation; frequent cases of childhood candidiasis have been reported [22]–[24].

Previous studies have reported mucous and cutaneous infections as the most common manifestation of pediatric candidiasis. Similarly, a study in Iran recovered *Candida* isolates predominantly from the oral cavity and stool of neutropenic patients suffering from leukemia [24]. However, in our study, the majority of the isolates were recovered from urine 121/150 (80.67%) and blood 15/150 (10.0%) specimens. Also, this contrasts the report from another medical center in Iran where the majority of *Candida* isolates among children were recovered from nails (39.5%) and blood (13.9%) infections [25]. Haddadi et al. reported that yeast isolates were the least recovered from urine specimens of neutropenic children [24]. Furthermore, out of the 498 clinical specimens investigated in a multicenter study, the majority of the *Candida* isolates from adults were recovered from vagina (32.5%) and oral cavity (24.3%) [26]. The observed heterogeneity might have resulted from the peculiarity of the subgroup and the underlying medical conditions of the patients studied.

*C. africana* and *C. dubliniensis* are emerging yeasts that attain clinical and epidemiological significance and, together with *C. albicans* form the *C. albicans* complex [26]. Discrimination between members of the complex is improbable using the conventional mycological tools. The molecular assay that involves amplification of the *HWP1* gene was among the earliest and reliable tools, that stands the test of time, to discriminate between the uncommon species of the complex [4]. Similar to the current study, *C. africana, C. dubliniensis*, and *C. albicans* were adequately identified using *HWP1* gene amplification [4],[9]–[11],[27]. In our study, *C. albicans* (94.23%) was the predominant species identified among the *C. albicans* complex which is in agreement with other studies [9]–[11],[13],[16],[21],[28]. We also identified both *C. africana* and *C. dubliniensis* among the urine isolates. Although *C. africana* initially appeared to be restricted to Africa and Europe and largely isolated from genital samples, it is now clear to have a worldwide distribution and recoverable from various clinical specimens [3],[12],[13],[20]. We found in our investigation that *C. africana* has a higher isolation rate (5.13%) than the *C. dubliniensis* (0.64%). This result corroborates the findings reported in Iran-where the frequency *C. africana* (3%) and *C. dubliniensis* (0%) was reported among 100 vaginal isolates of *C. albicans* species complex [10]–and a multicenter study that reported a higher frequency of *C. africana* (7.2%) than *C. dubliniensis* (2.9%), among *Candida* isolates recovered from 498 clinical specimens originated from various patient groups [26]. However, the higher isolation rate of *C. dubliniensis* over *C. africana* is being reported from other studies [9],[13],[19],[20]. Whereas *C. dubliniensis* is being predominantly recovered from oral and oropharyngeal lesions of HIV/AIDS patients, isolates were shown to be recovered from other patients suffering from cancer [14], cystic fibrosis [27], genital and respiratory tracts infections [9]–[13],[19],[20],[28], and from a gastric fluid sample [26]. The reason(s) for the disparate frequencies of isolation might be linked to factors such as the difference in the quality of sampling, the anatomic sites, the nature of the clinical sample, the underlying disease entities, racial or genotypic differences, and the immune status of the individuals involved.

Contrary to our findings, other studies that attempted to identify the cryptic species of *C. albicans* complex were able to identify, in addition to *C. albicans*, only *C. africana* [10],[16],[28] or *C. dubliniensis* [11],[27]. Interestingly, Gumral *et al*, evaluated vaginal *Candida* isolates using *HWP1* gene polymorphism and found neither *C. dubliniensis* nor *C. africana* among the *C*. *albicans* complex they investigated [29]. While any member of the complex can independently colonize its preferential anatomic sites, isolation of dual or multiple species have been reported from a single clinical sample [11],[24]. Similarly, we identified *C*. *albicans* in combination with *C. africana* in six of our urine isolates. An effort to discriminate species in mixed infection/colonization, especially in children, is very important for clinicians because each species tends to show a variable level of virulence and different susceptibility profile to antifungal agents.

## 5. Conclusions

Because they are not routinely sought for, the prevalence of *C. dubliniensis* and/or *C. africana* might be underestimated. However, it is evident that they are currently gaining both clinical and epidemiologic significance. Clinicians and scientists shall pay attention to and explore the significance of the cryptic species of *C*. *albicans* complex especially in pediatric settings. To our knowledge, we report the first case of *C*. *africana* candiduria among pediatrics in Tehran. Further studies are needed to support its clinical and pathologic significance in causing urinary tract infection among children.
